# The Self-Awareness Multilevel Assessment Scale, a New Tool for the Assessment of Self-Awareness After Severe Acquired Brain Injury: Preliminary Findings

**DOI:** 10.3389/fpsyg.2020.01732

**Published:** 2020-07-24

**Authors:** Umberto Bivona, Paola Ciurli, Giulia Ferri, Tiziana Fontanelli, Susanna Lucatello, Teresa Donvito, Dolores Villalobos, Laura Cellupica, Fabiana Mungiello, Paola Lo Sterzo, Amalia Ferraro, Eleonora Giandotti, Giorgio Lombardi, Eva Azicnuda, Carlo Caltagirone, Rita Formisano, Alberto Costa

**Affiliations:** ^1^Istituto di Ricovero e Cura a Carattere Scientifico (IRCCS) Santa Lucia Foundation, Rome, Italy; ^2^Department of Psychology, Faculty of Health Sciences, Camilo José Cela University, Madrid, Spain; ^3^Laboratory of Cognitive and Computational Neuroscience, Center for Biomedical Technology (Technical University of Madrid and Complutense University of Madrid), Madrid, Spain; ^4^Tor Vergata University, Rome, Italy; ^5^Università Telematica Niccolò Cusano, Rome, Italy

**Keywords:** severe acquired brain injury, anosognosia, self-awareness multilevel assessment, neurorehabilitation, functional deficit

## Abstract

Self-awareness (SA) is frequently impaired after severe acquired brain injury (sABI) and may lead to reduced subject’s compliance to treatment, worse functional outcome, and high caregiver distress. Considering the multifaceted nature of SA, a specific and effective assessment is crucial to address treatment of impairment of SA (ISA). Many tools can currently assess ISA; however, they have some important limits. In the present study, we proposed the Self-Awareness Multilevel Assessment Scale (SAMAS), a new scale for assessment of SA at different levels (i.e., *declarative*, *emergent*, and *anticipatory*) across all domains of functioning. The SAMAS has been designed to be administered by the cognitive/behavioral therapist with the involvement of a patient’s relative. Findings showed that the SAMAS allowed specifically assessing SA at a *declarative* level and on all possible functional domains. More interestingly, it seems also able to assess both *emergent* and *anticipatory* SA, thus overcoming some important limits of other current assessment methods. Our findings are consistent with a holistic perspective of the patient with sABI because thanks to the combined use of assessing tools, the SAMAS can provide an accurate diagnosis of ISA, thus better addressing the neurorehabilitation treatment and, accordingly, reducing the possible occurrence of its primary and secondary implications.

## Introduction

Self-awareness (SA), defined by Prigatano and Schacter (1991) as “*the capacity to perceive the ‘self’ in relatively ‘objective’ terms whilst maintaining a sense of subjectivity*,” is frequently impaired after a severe acquired brain injury (sABI) (Levy et al., 1998; Andersson and Finset, 2000; Gainotti and Marra, 2002), with a prevalence of impairment varying from 76 to 97% depending on the method of measurement adopted (Sherer et al., 1998).

Impaired self-awareness (ISA) has been associated to apathy or anosodiaphoria (Babinski, 1914; Tranel, 2002; Heilman and Harciarek, 2010; Gasquoine, 2016; Bivona et al., 2019), reduced subject’s compliance to treatment, worse functional outcome (Lam et al., 1988; Pollens et al., 1988; Ezrachi et al., 1991; Prigatano and Leathem, 1993; Fleming et al., 1996; Sherer et al., 1998, 2003; Prigatano and Wong, 1999; Bivona et al., 2014), and caregiver distress (Prigatano, 2005). Thus, a careful and early assessment of ISA after sABI is an important clinical issue.

However, some issues are still debated.

First: which is the functional architecture of SA? Crosson et al. (1989) posited a pyramidal model consisting of three interdependent and hierarchical levels: (a) *intellectual* awareness, that is, the subject’s ability to understand (mostly thanks to relatives’ or clinicians’ feedback) and refer that a function is impaired; (b) *emergent* awareness (a subsequent level of the former), the subject’s ability to recognize problems when they happen; and finally (at the top of the pyramid) (c) *anticipatory* awareness, that is, the ability to *anticipate* that a problem will occur because of a deficit (Crosson et al., 1989). Toglia and Kirk (2000), instead, proposed the Dynamic Comprehensive Model of Awareness (DCMA), which views the relationship between different aspects of metacognition and awareness as a dynamic process, rather than as a series of hierarchical levels (Toglia and Kirk, 2000). The DCMA differentiates between (a) *metacognitive awareness*, that is, knowledge of task characteristics and knowledge of one’s own capabilities (similarly to – even if broader than – the concept of *intellectual* SA of Crosson et al.’s model); and b) *online awareness* (activated during a task), which consists of the self-monitoring and recognition of errors (similarly to *emergent* SA of Crosson et al.’s model), as well as of the person’s appraisal of current task demands (comparable with *anticipatory* SA of Crosson et al.’s model). For the purpose of the present study, we will adopt the term “declarative” SA, referring to intellectual/metacognitive levels of SA.

Second: does SA affect different domains homogeneously? In fact, an individual may recognize some specific deficits (e.g., motor impairment), but being unaware of deficits in other domains (i.e., everyday problem solving or social situations) (Toglia and Kirk, 2000). In general, patients tend to show more severe ISA for behavioral and affective functions, moderate for cognitive functions, and less severe for motor and sensory functions (Sherer et al., 1998; Hart et al., 2003, 2004).

Third: how to best assess ISA after sABI? Several methods have been proposed: (a) clinical observation (Langer and Samuels, 2008), even by structured rating scales such as the “Clinician’s Rating Scale” (Prigatano and Klonoff, 1998); (b) structured and semi-structured interviews, such as the “Self-Awareness Deficits Interview” (SADI; Fleming et al., 1996) or the “Self-Regulation Skills Interview” (Ownsworth et al., 2000); (c) by comparing patient’s self-assessment and their performance on neuropsychological tests, such as the “Awareness Interview” (Anderson and Tranel, 1989) and the “Assessment of Awareness of Disability” (Tham et al., 1999); and (d) the comparison between patient’s self-report and clinician/relative’s report, such as the “Patient Competency Rating Scale” (PCRS; Prigatano et al., 1986), the “Awareness Questionnaire” (Sherer et al., 1998), and the Head Injury Behavior Scale (Godfrey et al., 1993).

However, these methods have some limits. For example, they cannot be administered to patients who suffer from aphasia, as well as from severe memory deficits or reduced reasoning and judgment abilities. Moreover, to our knowledge, currently used inventories and interviews can assess solely *declarative* awareness. In fact, to assess *emergent* and *anticipatory* awareness, patient’s performance has to be evaluated in a variety of situations by a trained professional (Barco et al., 1991). In this regard, the limits of studies investigating *emergent* (Ownsworth et al., 2000, 2002; Abreu et al., 2001; O’Keeffe et al., 2007; Krasny-Pacini et al., 2014; Dockree et al., 2015) or *anticipatory* (Fleming et al., 1996; O’Keeffe et al., 2007) SA after sABI are the use of tasks sensitive only to some specific cognitive or behavioral functions. Importantly, as *anticipatory* awareness allows the implementation of correct future behavior, it needs to be *objectively* assessed. Indeed, the *offline* collection of the patient’s report itself, reflecting just a *declarative* awareness on possible future difficulties in relation to the post-injury difficulties, is not a reliable method: only an external report (e.g., by an informal caregiver of the care recipient) about the patient’s real behavior during the activities of daily life can evidence more reliably that the patient has *actually* gained anticipatory SA (Stuss, 1991; Flashman et al., 1998).

Given this background, the aim of the present study was to examine the validity of the Self-Awareness Multilevel Assessment Scale (SAMAS) to assess ISA after sABI. The SAMAS assesses different *levels* of SA (i.e., declarative, emergent, and anticipatory) across all *domains of functioning* (i.e., physical, cognitive, emotional/behavioral). It has been designed to be completed by the cognitive/behavioral therapist, with the involvement of the patients’ relative, as well as of other members of the inter-professional neurorehabilitation team, when necessary. Furthermore, to examine the potential added value of SAMAS to assess ISA after sABI in respect to extant tools, the PCRS and the SADI, two of the most currently used scales to assess ISA in this population, were also administered.

## Materials and Methods

### Participants

Twenty-five patients with sABI, consecutively admitted to the Post-Coma Unit of Santa Lucia Foundation in Rome (Italy) from March 2018 to September 2019, were recruited. The study was approved by the local Ethics Committee, and all participants were included in the study after providing their (or by one legal surrogate) informed consent.

Patients with sABI were recruited according to the following *inclusion* criteria: (a) age ≥ 16 years; (b) diagnosis of severe ABI (Glasgow Coma Scale score ≤ 8 in the acute phase); (c) score at the Level of Cognitive Functioning Scale ≥ 6, with inclusion of the patient according to the judgment of the neuropsychologist involved in the study; d) capacity to undergo a formal psychological evaluation; (e) availability of informed consent.

Exclusion criteria for patients recruited in this study were (a) history of drug and alcohol addiction, (b) psychiatric diseases, and (c) repeated sABI and/or other neurological disorders.

Socio-demographic and clinical characteristic of patients were 19 males and 6 females, with a mean age of 43.4 years (*SD* = 16.1); mean educational level of 12.4 years (*SD* = 4.2); time since injury from 45 to 472 days, with a mean of 131.2 days (*SD* = 97.1); and etiology of sABI: Traumatic Brain Injury (TBI) (*n* = 15), stroke (*n* = 9), and other causes (*n* = 1).

### Measures

#### Functional Assessment

A functional assessment was performed by means of the following scales: (1) Glasgow Outcome Scale (Jennett and Bond, 1975); (2) Level of Cognitive Functioning (LCF) scale (Hagen et al., 1972); (3) Disability Rating Scale (Rappaport et al., 1982).

#### Self-Awareness Assessment

##### Gold Standard Measure of Self-Awareness

To date, no measures useful to assess concurrent validity regarding *emergent* and *anticipatory* SA are available; thus, evaluation of both levels of SA requires observations of the patient’s task performance in a variety of situations, accompanied by timely questions by a trained professional (Barco et al., 1991). Accordingly, in line with other studies who underline the role of the clinician as a rater of the level of SA (Fleming et al., 1996), in the present study we adopted as a gold standard measure of SA the clinical judgment of a neuropsychologist (P.C.) expert for around 30 years in the field of severe ABI. It is worth noting that she judged SA in the context of a complete neuropsychological assessment performed by herself, which enhanced the reliability of her global assessment of SA. Indeed, by a careful observation of the patients’ behavior and self-monitoring during the test administration, completed by a clinical interview to the patients and their caregiver, and by information gathered together with other professional of the rehabilitation team (i.e., nurses, occupational therapists, physiotherapists), the neuropsychologist assessed at best all the levels of SA taken into account in the present work, in all the possible domains.

In particular, for each level, the neuropsychologist assessed patients in blind with respect to the other professional included in the study, scoring between 0 (i.e., “good SA”) and 4 (“severe ISA”) on each one of the several *domains* of interest (i.e., motor/sensitive, cognitive, behavioral/affective, and other) and *levels* of SA (i.e., declarative, emergent, and anticipatory SA). Accordingly, the maximum possible score on each domain was 12, being the maximum possible total score equal to 36.

##### Self-Assessment Multilevel Scale

In the present study, we developed this new scale with the main purpose of assessing, by a single and comprehensive tool, the aforementioned different levels of SA (i.e., *declarative*, *emergent*, and *anticipatory*) along with the following domains: motor, cognitive, psycho-behavioral, and others (i.e., phoniatric, dysphagic). Within each level, the scores for each domain can range from 0 (i.e., “good SA”) to 2 (“relevant ISA”). In particular, score 0 for each level and domain is index of patients’ ability to spontaneously recognize their possible difficulties; score 1, index of their ability to recognize possible difficulties only *after receiving a cue* by the therapist; and score 2, index of a *severe impairment* in recognizing their possible difficulties even after such cue. When a patient does not present with any problem in one or more of the domains, SAMAS in that or those domains is scored as “not applicable” (see Supplementary Annex for more details on the scale).

The *declarative* level consisted of two items: patient’s recognition of the presence of current difficulties, and the functional implications of such difficulties. The *emergent* level is composed of one item referring to the patient’s *online* recognition of difficulties, if and when they occur in each domain. Finally, as for the *anticipatory* level, the SAMAS contains five items: the patient’s ability to recognize the problematic nature of a task with respect to his/her own deficits; the patient’s ability to set realistic goals in relation to his own difficulties; the patient’s expression of strategies to avoid having difficulties; the patient’s effective use of such strategies; the patient’s ability to generalize such strategies (when they are used) to all the contexts in which he/she acts.

The SAMAS can be completed by a cognitive/behavioral therapist as soon as he/she has a clear and complete picture of the patients’ SA at the different levels and on the different domains. In particular, in the present study the SAMAS has been completed by a cognitive/behavioral therapist within 30 min maximum, and within 10 observation sessions in all cases. The SAMAS has been conceived to be completed with the support of the physiotherapist and informal caregivers, to obtain a clear and complete picture of the patients’ SA. In particular, both physiotherapists and informal caregivers allowed verifying two main aspects: (a) the *emergent* SA even in other contexts (for instance, if the patients are able to recognize motor difficulties when they occur during the sessions of physiotherapy, or in their hospital room or at home); (b) the patients’ *anticipatory* SA *beyond its declarative* level, that is, verifying if it actually corresponds to a *real anticipatory* level of SA, thanks to an external report on patients’ behaviors outside the cognitive/behavioral setting. Accordingly, in assessing both *emergent* and *anticipatory* SA, each cognitive/behavioral therapist involved in the present study scored the SAMAS only after having collected and accurately weighed the information reported by both physiotherapists and patients’ informal caregivers.

##### Patient Competency Rating Scale

The PCRS (Prigatano et al., 1986; Ciurli et al., 2010) is a 30-item self-report questionnaire that requires patients and their relatives to make an independent judgment of perceived degree of competency demonstrated in several behavioral, cognitive, and emotional situations. To assess declarative SA, it is important to consider both the patient self-report and the magnitude of the difference between patients’ report and the relative report of patients’ functional competency, that is, the PCRS discrepancy score (PCRS-DS) (Prigatano et al., 1990; Prigatano, 2014; Bivona et al., 2019). Higher PCRS-DS mean higher ISA.

##### Self-Awareness Deficits Interview

The SADI (Fleming et al., 1996) is a clinician-rated measure in a semi-structured interview format. It includes items addressing three domains: SA of deficits, SA of the functional implication of the deficits, and ability to set realistic goals. Higher scores mean more impaired SA.

### Procedure

After admission to the Post-Coma Unit of the Santa Lucia Foundation IRCCS, a neurorehabilitation hospital in Rome, the whole SA assessment was conducted to all patients as soon as they were diagnosed by our expert neuropsychologist (P.C.) as emerged from the level 5 of LCF scale, that is, when their responses, even if they might have been incorrect because of memory problems, were appropriate to the situation; or when they showed beginning immediate awareness of personal situation; or when they no longer wandered and were, even inconsistently, oriented to time and place.

Within the same week, for each patient, the same neuropsychologist, a cognitive/behavioral therapist, and a clinical psychologist completed his/her assessment in blind with respect to each other, as follows: (a) the neuropsychologist began, in a quiet room, the administration of the neuropsychological test battery and, by observing the patients in that context, with the support of the patient’s informal caregiver, physiotherapist, and nurses of the Post-Coma Unit, she completed the assessment of SA (i.e., the gold standard assessment in the present study); (b) a cognitive/behavioral therapists (G.F., S.L., L.C., F.M., or P.L.) completed both the SADI and the SAMAS in the context of the cognitive/behavioral neurorehabilitation setting (i.e., in another room of the Post-Coma Unit); (c) a clinical psychologist (T.D. or G.L.) administered, in a third room, the PCRS to each patient and (separately) to his/her caregiver.

### Statistical Analysis

Data analysis was carried out using SPSS software (version 22). Preliminarily, we described the study variables in terms of means and SDs to illustrate the characteristics of the patients.

To investigate whether participants’ score on SAMAS predict clinical judgment of SA, the forward linear regression model was applied. In particular, the specific aim of the study is to explore whether SAMAS can improve the clinical diagnosis of ISA in respect to some of the extant tools. Indeed, SAMAS directly assesses two SA factors (i.e., *anticipatory* and *emergent* SA) that are not fully taken into account by currently used tools. Accordingly, in the regression model, the clinical judgment (score range 1–4) was entered in the model as dependent variable and global scores on SAMAS, PCRS-DS, and SADI were entered as independent variables. Therefore, four forward linear regression analyses were run in which the independent variables were those indicated above. As for the dependent variables, they were as follows: in the first analysis, the *global* clinical judgment of SA; in the second, the clinical judgment of *declarative* SA; in the third, the clinical judgment of *emergent* SA; in the fourth, the clinical judgment of *anticipatory* SA, respectively. Pearson’s *r* correlations were executed to investigate the association between the clinical judgment on each of the three dimensions of SA and the score on the corresponding subscale of the SAMAS. Pearson’s *r* correlations were also performed to investigate the association between participants’ global scores on SAMAS, PCRS-DS, and SADI.

## Results

Descriptive statistics for the sample including demographic, functional, and SA are reported in Table 1.

**TABLE 1:** Demographic, functional, and self-awareness variables of the sample (*N* = 25).

	Mean	*SD*	Range
**Demographic variables**			
Age	43.4	16.1	13–66
Educational level	12.4	4.2	8–24
Time since injury (days)	131.2	97.1	45–472
**Functional variables**			
GOS	3.2	0.4	3–4
LCF	6.9	0.3	6–7
DRS	10.5	7.1	0–21
**Self-awareness variables**			
Gold standard	10.4	10.6	0–33
SAMAS	18.4	13.9	0–48
PCRS-DS	–0.2	9.7	–22 to 24
SADI	3.8	2.8	0–9

*GOS, Glasgow Outcome Scale; LFC, Level of Cognitive Functioning Scale; DRS, Disability Rating Scale; SAMAS, Self-Assessment Multilevel Scale; PCRS-DS, Patient Competency Rating Scale discrepancy score; SADI, Self-Awareness Deficit Index.*

### SA Scores Predicting the Global Clinical Judgment of SA

In the first step of this analysis, the SAMAS total score entered the regression equation [*F*(1, 24) = 25.9; *p* < 0.001; *R*^2^ = 0.53] with a positive correlation with the dependent variable (Beta = 0.73; *t* = 5.09; *p* < 0.001). This shows that higher scores on SAMAS are associated with worse SA according to the global clinical judgment. In the second step, the score on the PCRS-DS significantly contributed to the model [*R*^2^ change = 0.10; *F*(2, 24) = 18.9; *p* < 0.001], also in this case with a positive correlation with the dependent variable (Beta = 0.35; *t* = 2.48; *p* = 0.021). This documents that higher PCRS-DS are associated with global clinical judgment of more severe ISA. SADI score, instead, did not enter the regression equation (Beta = −0.29; *t* = −0.15; *p* > 0.80) (Table 2).

**TABLE 2:** Results of the forward linear regression analysis performed to investigate whether participants’ score on SAMAS predicted *global clinical judgment* of self-awareness.

Model	*B*	Beta	Standard error	*t*	*P*-value
**1**					
Constant	0.020		2.717	0.007	>0.90
SAMAS total score	0.696	0.728	0.137	5.092	<0.001
**2**					
Constant	0.451		2.461	0.183	>0.80
SAMAS total score	0.553	0.579	0.136	4.067	0.001
PCRS-DS total score	0.413	0.354	0.166	2.485	0.021

*Dependent variable: global clinical judgment of SA; SAMAS, Self-Assessment Multilevel Scale; PCRS-DS, Patient Competency Rating Scale discrepancy score.*

### SA Scores Predicting the Clinical Judgment of Declarative SA

Results of this analysis are similar to the results of analysis presented previously. In the first step, the SAMAS total score entered the regression equation [*F*(1, 24) = 23.2; *p* < 0.001; *R*^2^ = 0.50] with a positive correlation with the criterion (Beta = 0.71; *t* = 4.82; *p* < 0.001). This shows that higher scores on SAMAS are associated with more severe impairment in declarative SA according to the clinical judgment. In the second step the PCRS-DS score also entered the regression equation [*R*^2^ change = 0.17; *F*(2, 24) = 24.3; *p* < 0.001], showing a positive correlation with the dependent variable (Beta = 0.47; *t* = 3.62; *p* = 0.002). This documents that worse declarative SA according to PCRS-DS are associated with global clinical judgment of more severe declarative SA. Also in this case, SADI score did not significantly contribute to the model (Beta = 0.15; *t* = 0.85; *p* > 0.30) (Table 3).

**TABLE 3:** Results of the forward linear regression analysis performed to investigate whether participants’ score on SAMAS predicted the clinical judgment of *declarative* self-awareness.

Model	*B*	Beta	Standard error	*t*	*P*-value
**1**					
Constant	–0.481		0.920	–0.523	>0.60
SAMAS total score	0.223	0.709	0.046	4.816	<0.001
**2**					
Constant	–0.291		0.747	–0.389	>0.70
SAMAS total score	0.160	0.509	0.041	3.874	0.001
PCRS-DS total score	0.183	0.475	0.050	3.621	0.002

*Dependent variable: clinical judgment of declarative SA; SAMAS, Self-Assessment Multilevel Scale; PCRS-DS, Patient Competency Rating Scale discrepancy score.*

### SA Scores Predicting the Clinical Judgment of Emergent SA

Results of this analysis show that the only independent variable entering the regression equation was the SAMAS score [*F*(1, 24) = 27.3; *p* < 0.001; *R*^2^ = 0.54] with a positive correlation with the criterion (Beta = 0.74; *t* = 5.22; *p* < 0.001). This result documents that higher scores on SAMAS are associated with more reduced emergent SA according to the clinical judgment. In this case, neither PCRS-DS (Beta = 0.27; *t* = 1.84; *p* = 0.08) nor SADI scores (Beta = 0.01; *t* = 0.05; *p* > 0.90) significantly contributed to the model (Table 4).

**TABLE 4:** Results of the forward linear regression analysis performed to investigate whether participants’ score on SAMAS predicted the clinical judgment of *emergent* self-awareness.

Model	*B*	Beta	Standard error	*t*	*P*-value
**1**					
Constant	–0.120		0.853	–0.141	>0.80
SAMAS total score	0.224	0.737	0.043	5.225	<0.001

*Dependent variable: clinical judgment of emergent SA; SAMAS, Self-Assessment Multilevel Scale; PCRS-DS, Patient Competency Rating Scale discrepancy score.*

### SA Scores Predicting the Clinical Judgment of Anticipatory SA

Results of this analysis also show that SAMAS score was the only independent variable entering the regression equation [*F*(1, 24) = 19.3; *p* < 0.001; *R*^2^ = 0.46], also in this case showing a positive correlation with the dependent variable (Beta = 0.68; *t* = 4.39; *p* < 0.001). This result indicates that higher scores on SAMAS are associated to worse clinical judgment of anticipatory SA. Both PCRS-DS (Beta = 0.29; *t* = 1.77; *p* = 0.09) and SADI scores (Beta = 0.03; *t* = 0.16; *p* > 0.80) were excluded from the regression model (Table 5).

**TABLE 5:** Results of the forward linear regression analysis performed to investigate whether participants’ score on SAMAS predicted the clinical judgment of *anticipatory* self-awareness.

Model	*B*	Beta	Standard error	*t*	*P*-value
**1**					
Constant	0.621		1.126	–0.552	>0.50
SAMAS total score	0.249	0.676	0.057	4.395	<0.001

*Dependent variable: clinical judgment of anticipatory SA; SAMAS, Self-Assessment Multilevel Scale; PCRS-DS, Patient Competency Rating Scale discrepancy score.*

### Correlations Between the Clinical Judgment on Each of the Three Dimensions of SA and the Score on the Corresponding Subscale of the SAMAS

Results of Pearson’s *r* correlation analyses show a highly significant positive correlation between the *declarative* score obtained on the SAMAS and the clinical judgment of declarative SA (*r* = 0.67; *p* < 0.001) as well as between score for the *anticipatory* items of the SAMAS and the clinical judgment of anticipatory SA (*r* = 0.62; *p* < 0.001). As for the correlation between *emergent* score obtained on the SAMAS and the clinical judgment of emergent SA in this case, instead, we found a tendency toward a significant effect (*r* = 0.33; *p* = 0.053).

### Correlations Between SAMAS, PCRS-DS, and SADI Scores

Results from these analyses document that participants’ global scores on SAMAS, PCRS-DS, and SADI are significantly correlated with each other. In particular, scores on SAMAS positively correlated with scores on both PCRS-DS (*r* = 0.42; *p* = 0.036) and SADI (*r* = 0.66; *p* < 0.001). In turn, scores on PCRS-DS and SADI also showed a significant positive correlation (*r* = 0.52; *p* = 0.008). These results clearly indicate that scores on the three scales are congruently associated.

## Discussion

This study aimed at investigating the validity of a new tool, the SAMAS, to assess ISA in people with sABI. First, ISA was assessed by an expert neuropsychologist who rated a clinical judgment on a 4-point scale on the *declarative*, *anticipatory*, and *emergent* dimensions of SA. Then, regression analyses were performed to examine the predictive value of SAMAS score on the clinical judgment earlier, taking into account the weight of the PCRS-DS and SADI score. Main results show that SAMAS scores significantly predicted all dimensions of SA. Interestingly, the SAMAS score was the unique variable entering the regression equation in the analyses, including the clinical judgment of *anticipatory* and *emergent* ISA as dependent variables. Moreover, results document a highly significant positive correlation between the *declarative* score obtained on the SAMAS and the clinical judgment of declarative SA as well as between score for the *anticipatory* items of the SAMAS and the clinical judgment of anticipatory SA, whereas for the correlation between *emergent* score obtained on the SAMAS and the clinical judgment of *emergent* SA, we found a tendency toward a significant effect.

These results indicate that SAMAS is able to *specifically* and *broadly* assess both emergent and *(actual)* anticipatory SA. Indeed, although SAMAS showed significant positive correlations with both PCRS-DS and SADI, thus indicating that high scores on the three scales coherently outline low global levels of SA, as from results of regression analyses its score was independently associated to the clinical judgment on anticipatory and emergent SA (i.e., PCRS-DS and SADI scores did not enter the regression equation). This represents an important and innovative contribution of the present study allowing overcoming some important limits of other current methods of assessment of SA. Indeed, the extant tools that assessed *emergent* SA (Ownsworth et al., 2000, 2002; Abreu et al., 2001; O’Keeffe et al., 2007; Krasny-Pacini et al., 2014; Dockree et al., 2015) in the field of ABI utilize only a few number of specific tasks, on limited cognitive or behavioral domains. Conversely, the SAMAS (*emergent* section) has been completed by the cognitive/behavioral therapists after an accurate *online* observation of patients’ behavior and report during several performances within the neurorehabilitation context, as well as thanks to collecting information from the patients’ physiotherapist and caregiver in other contexts (for instance, during the sessions of physiotherapy, or in the hospital room or at home). These series of measures correlated with the clinical judgment of our expert neuropsychologist who, in parallel and blindly, assessed *emergent* SA within the neuropsychological assessment context. In fact, this can be considered an important index of concurrent validity. In this regard, as reported previously, it should be noted that although we found an association between the score on emergent subscale of SAMAS and the clinical judgment of emergent SA (*r* = 0.33; *p* = 0.053), such an association only approached statistical significance, likely as a result of the relatively limited sample size.

Similarly, the studies in the literature which aimed at assessing *anticipatory* SA (Fleming et al., 1996; O’Keeffe et al., 2007) considered, as a matter of fact, only its *declarative* aspects, without providing a confirmation of a *real* anticipatory SA, such as, for instance, the fact that patients avoided dangerous or dysfunctional behaviors in their daily life. Even in this case, SAMAS allowed overcoming this limit because therapists completed the *anticipatory* section of the scale only when patients’ report were consistent with the parallel interview to their physiotherapists and informal caregivers regarding their real behaviors outside the cognitive/behavioral neurorehabilitation context and, more generally, during daily life. Only this comparison allowed therapist claiming whether the patients effectively gained an *actual anticipatory* SA, *beyond its declarative aspect*.

A final comment should be deserved to the finding of a highly significant correlations we found between the three subscales of SAMAS.

We would underline that our study must be considered just as preliminary, owing to the limited sample size, as well as to not having investigated the inter-rater reliability of the SAMAS between the different cognitive/behavioral therapists who took part in the study. Nevertheless, taken together, our findings are consistent with the main aim of the present study that is proposing a new clinical tool to deeply and quantitatively assess SA at its different levels (at least according to the main theoretical models) (Crosson et al., 1989; Toglia and Kirk, 2000), and on the possible functional domains.

## Conclusion

In conclusion, it is worth noting that this preliminary study is part of a study still in progress because, by enlarging the sample size, we are aiming at investigating also the inter-rater reliability of the SAMAS.

However, our current results suggest that the SAMAS can be conceived as a useful scale to broadly assess SA and, in particular, to quantify some relevant information on patients’ levels of SA, which usually remain only as a part of a qualitative clinical observation. Indeed, the great advantage of SAMAS is that it allows the cognitive/behavioral therapist to systematically quantify what is usually observed within the rehabilitation setting regarding the different *levels* of self-awareness on each functional *domain*. Accordingly, a careful use of SAMAS would allow a better monitoring of ISA within the neurorehabilitation process, as well as a more reliable comparison between different professionals in rehabilitation. In particular, to date, it seems to be the only tool in the literature that allows the assessment of *emergent* and (really, not just declaratively) *anticipatory* SA.

However, we would also underline that the SAMAS can be a thorough and effective assessing tool of ISA *as long as* (a) it is completed within the context of an accurate *clinical observation*, (b) if it is accompanied by an accurate *interview to the informal caregivers*, and (c) if it is supported by the necessary *information gained by the other members of the inter-professional neurorehabilitation team*; accordingly, an adequate experience with team work is mandatory to achieve a correct coding of the SAMAS. Moreover, to better assess *declarative* level of SA, we also recommend the combined use of the SAMAS with some of the traditional questionnaires or interviews, to enhance the reliability of all measures used. Indeed, only a holistic approach to the patient with sABI, thanks to the combined use of clinical observation, interviews and scales, can allow obtaining an early and accurate diagnosis of ISA. Accordingly, it is possible also to better address the ISA treatment and reduce the possible occurrence of its primary (e.g., poor motivation and compliance; hostility toward therapy, and risk of failure of rehabilitation) and secondary (e.g., poor ability of risk evaluation, ineffective behaviors, poor social and work re-entry) implications, that so often make ISA an everlasting problem not only for the patients but sometimes even more for their whole family and social systems.

## Data Availability Statement

The raw data supporting the conclusions of this article will be made available by the authors, without undue reservation.

## Ethics Statement

The studies involving human participants were reviewed and approved by the Comitato Etico of the IRCCS Fondazione Santa Lucia di Roma (It) – Santa Lucia Foundation local Ethics Committee. Written informed consent to participate in this study was provided by the participants’ legal guardian/next of kin.

## Author Contributions

UB, GF, and TF conceived the study. AC designed and supervised the project and analyzed the data. PC collected the data as a gold standard. GF, SL, LC, FM, and PL collected the data on the SAMAS and SADI scales. TD, TF, AF, and EG collected the data on PCRS scale. DV, GL, and EA managed the database and contributed to the general organization and realization of the project. CC and RF supervised and gave an intellectual contribution to the project. UB wrote the first draft of the manuscript. All authors listed have made a substantial, direct and intellectual contribution to the work, and approved it for publication.

## Conflict of Interest

The authors declare that the research was conducted in the absence of any commercial or financial relationships that could be construed as a potential conflict of interest.
