# Supplementary material for: The Self-Awareness Multilevel Assessment Scale, a New Tool for the Assessment of Self-Awareness After Severe Acquired Brain Injury: Preliminary Findings
Source: Front Psychol. 2020 Jul 24;11:1732. doi: 10.3389/fpsyg.2020.01732 (PMC7393213; doi:10.3389/fpsyg.2020.01732)
Supplement: Supplementary file 1 [file Data_Sheet_1.pdf]

Self-Awareness Multilevel Assessment Scale

U. Bivona*, P. Ciurli, G. Ferri, T. Fontanelli, S. Lucatello, T. Donvito, D. Villalobos, L. Cellupica, F. Mungiello, P. Lo Sterzo, A. Ferraro, E. Giandotti, G. Lombardi, E. Azicnuda, C. Caltagirone, Rita Formisano, and A. Costa

**u.bivona@hsantalucia.it*

DECLARATIVE self-awareness	Yes (0)	With cue (1)	No (2)	N.A
Patient **recognizes the presence of current difficulties** (**specify** the difficulties already known to the patient on the **basis of the NPS report**, highlighted by **clinical observation** or **reported by the caregiver**):				
Motor: ________________	❒	❒	❒	❒
Cognitive: ________________	❒	❒	❒	❒
Psycho-behavioural: ________________	❒	❒	❒	❒
Others (specify which ones): ________________ ________________	❒	❒	❒	❒
Patient recognizes the **functional implications** of one's difficulties:				
Motor	❒	❒	❒	❒
Cognitive	❒	❒	❒	❒
Psycho-behavioural	❒	❒	❒	❒
Others ________________	❒	❒	❒	❒

EMERGENT self-awareness	Yes (0)	With cue (1)	No (2)	N.A
**When they occur,** patient **recognizes the presence of difficulties:**				
Motor (specify): ________________	❒	❒	❒	❒
Cognitive (specify): ________________	❒	❒	❒	❒
Psycho-behavioural (specify): ________________	❒	❒	❒	❒
Others (specify): ________________ ________________	❒	❒	❒	❒

ANTICIPATORY self-awareness	Yes (0)	With cue (1)	No (2)	N.A
Patient is able to **recognize the problematic nature of a task** with respect to his own deficits at the level:				
Motor	❒	❒	❒	❒
Cognitive	❒	❒	❒	❒
Psycho-behavioural	❒	❒	❒	❒
*Others*____	❒	❒	❒	❒
Patient is able to **set realistic goals** in relation to his own difficulties at level:				
Motor	❒	❒	❒	❒
Cognitive	❒	❒	❒	❒
Psycho-behavioural	❒	❒	❒	❒
*Others*____	❒	❒	❒	❒
Patient **declares the need for strategies** to avoid having difficulties at the level:				
Motor	❒	❒	❒	❒
Cognitive	❒	❒	❒	❒
Psycho-behavioural	❒	❒	❒	❒
*Others*____	❒	❒	❒	❒
Patient **effectively uses** strategies to avoid having difficulty at the level:				
Motor	❒	❒	❒	❒
Cognitive	❒	❒	❒	❒
Psycho-behavioural	❒	❒	❒	❒
*Others*____	❒	❒	❒	❒
Using strategies in the rehabilitation context, patient **is able to generalize to all the contexts in which he/she acts**, at the level:				
Motor	❒	❒	❒	❒
Cognitive	❒	❒	❒	❒
Psycho-behavioural	❒	❒	❒	❒
*Others*____	❒	❒	❒	❒
